# How to Live an Hundred Years

**Published:** 1857-03

**Authors:** 


### HOW TO LIVE AN HUNDRED YEARS.

Hufeland calls the stomach *Atria mortis*, the entrance-hall of death, and says without a good stomach it is impossible to attain a great age. All have naturally good stomachs, that is, good digestion, but it is ruined early by improper feeding as to time, quality, quantity, and mode of preparation. Therefore a large portion of the *Journal* will be occupied with statements in reference to these items. Substantial, nourishing food, properly prepared and well digested, these three are the great essentials of a long and healthy life. I will here give two examples, full of instruction, highly encouraging, and well worthy of imitation by all who would like to live in the full enjoyment of health, and all the faculties of mind and body, for a hundred or a hundred and fifty years or more. The first shows how an injured stomach and constitution may be repaired; the second how they may remain in perfect health for a hundred and fifty years, and all by the proper management of eating and drinking.

Lewis Cornaro, an Italian nobleman of wealth, by intemperance and debauchery made a wreck and ruin of his fortune and constitution at the early age of forty years. His physicians, considering his habits inveterate, informed him that restoration was impossible, and with characteristic recklessness he resolved that if he had to die, he would abandon himself to the fullest indulgences, and thus get all the good possible out of the short remnant of life before him. But some circumstance shortly occurred which induced him to reverse his decision, and experiment on the possibility of disappointing his doctors and his heirs, and living to a good old age. This he attempted at once by means of his food and drink alone. He began by eating and drinking very little, and found that his health improved. Sometimes he would eat more, then less, until he discovered what amount of food was most suitable for him, which was twelve ounces of solid food, and thirteen ounces of fluid, every twenty-four hours. At length his health became so good, that his friends suggested to him, that now he was so hearty and well, there was no longer any necessity for such a strict allowance, and that if he ate and drank a little more it would be of advantage to him. He replied that he was now well, and had continued well for some years on this allowance, that he could not be better, and that he had no disposition to run any unnecessary risks, nor to make hazardous experiments, and that as he had regained his title and estates, and his health too, he now wished greatly to preserve the last, that he might long enjoy the others. However, he was at length induced to gratify his friends, and increased his food to fourteen, and his drink to sixteen ounces a day; and said he:

> "Scarcely had I continued this mode of living ten days, when I began, instead of being cheerful and lively as before, to become uneasy and dejected, a burden to myself and to others. On the twelfth day I was seized with a fever of such violence, that for thirty-five days my life was despaired of. But by the blessing of God, and my former regimen, I recovered; and now, in my eighty-third year, I enjoy a happy state of both body and mind. I can mount my horse unaided. I climb steep hills. When I return home from a private company, or the senate, I find eleven grand-children, whose education, amusements, and songs, are the delight of my old age. I myself often sing with them, for my voice is clearer and stronger than it was in my youth; and I am a stranger to those peevish and morose humors which so often fall to the lot of old age."

In the latter years of his life he published an "*Earnest Exhortation*," which he closes by saying, "Since length of days abounds with so many blessings and favors, and I happen to be one of those who have arrived at that state, I cannot but give my testimony in favor of it; and I assure you all that I really enjoy more than I express, and that I have no other reason for writing, but that of demonstrating the great advantages which arise from longevity, to the end that their own conviction may induce them to observe those excellent rules of temperance and sobriety. And therefore I never cease to raise my voice, crying out to you, May your days be long, *that you may be the better servants to the Almighty.*"

When about to die, he raised his eyes and exclaimed, with great animation, "Full with joy and hope, I resign myself to thee, most merciful God!" He then disposed himself with dignity, and closing his eyes, as if about to slumber, gave a gentle sigh, and expired, in his ninety-ninth year, A. D. 1565.

If a systematic life of temperance has given sixty years additional to a broken-down constitution of forty, it becomes almost a crime for an invalid under fifty years of age not to avail himself of the trial.

This was a case where the energies of the stomach have been restored by temperance in eating and drinking, and remaining in their integrity for more than half a century thereafter; and what has been, may be again.

The next example shows that the stomach is made, *in modern times* too, to last a hundred and fifty years.

Thomas Parr, of Shropshire, England, when a hundred and twenty years old, married a widow for his second wife, who lived with him twelve years, and who stated that, during that time, he never betrayed any signs of age or infirmity. The King of England having heard of him, invited him to London in his hundred and fifty-second year. He was treated in so royal a manner at court, and his mode of living was so totally changed, that he died soon after, in 1635, aged one hundred and fifty-two years and nine months, proven by public documents. His body was examined by Dr. Harvey, who "found his internal organs in the most perfect state, nor was the least symptom of decay found in them. His cartilages, even, were not ossified, as is the case in all old people. The smallest cause of death had not settled in his body; and he died merely of plethora, because he had been too well fed."

This man was a farm-servant, and had to maintain himself by daily labor, consequently he must have lived on plain food, and not over-abundant; and the simple fact that at his death his stomach was in a healthy condition, proves conclusively its capabilities of duration, working healthfully to the last. And there can be no reason, in the nature of things, why the human stomach may not be preserved in its integrity, as a general rule, to a like old age.

I trust no reader will attempt to live on common allowance, on his own responsibility—he should consult with his family physician; for age, sex, condition in life, occupation, materially modify the amount of food requisite for the wants of the system.

